# The Workmen's Compensation Act, 1897

**Published:** 1898-01-22

**Authors:** 


### THE WORKMEN'S COMPENSATION ACT, 1897.

A matter of considerable interest to medical practitioners in regard to the payment for certificates under the Workmen's Compensation Act has been raised by Mr. Gibson Bowles in a letter to the *Times*. Among the provisions of this Act is one to the effect that if a workman receiving a weekly allowance is dissatisfied with the certificate given by the medical man employed he may submit himself for examination "to one of the medical practitioners appointed for the purposes of this Act, as mentioned in the second schedule to this Act," and his certificate shall be conclusive evidence as to his condition. Now, Mr. Bowles says that there is no provision in the Act for the payment of this medical practitioner. It should be noted, however, that paragraph 13 of schedule 2 says that "the Secretary of State may appoint legally qualified practitioners for the purpose of this Act, and any committee, arbitrator, or judge may, subject to regulations made by the Secretary of State and the Treasury, appoint any such practitioner to report on any matter which seems material to any question arising in the arbitration; and the expense of any such medical practitioner shall subject to Treasury regulations be paid out of moneys to be provided by Parliament." Mr. Bowles maintains that "this paragraph seems to give no authority whatever for the payment out of moneys to be provided by Parliament of any expense incurred in consequence of a workman submitting himself to examination." Moreover, he says that the man's condition cannot be considered to be a matter material to any question "arising in the arbitration," for a workman "receiving weekly payment under this Act" is one who is affected by an arbitration already completed and being carried out. We take it, however, that the medical practitioner referred to is appointed "for the purpose of this Act," and not for the purpose of one particular clause, and that the words "the expense of any such medical practitioner shall," &c., refer not only to the preceding sentence but to all the purpose of this Act. Moreover, it is not quite correct to say that in the case of a workman "receiving weekly payment" the arbitration is "already completed and being carried out," because in the body of the Act, section 3, it is said that "if any question arises in any proceedings . . . . as to the amount or *duration* of compensation," &c., it may be settled by arbitration, and the duration of compensation depends absolutely on the medical certificates. We have no doubt then that if a workman is not satisfied with the certificate given by the medical practitioner employed, he can bring the matter again to arbitration, and that in that case the State practitioner will be paid out of funds provided by Parliament; and in regard to the other point we think that much will depend upon the "Treasury regulations," subject to which the expense of the practioner is to be paid. The matter, however, is one of much interest, and we cannot but agree that the wording of the Act is far from being as clear as it ought to be.

